# In Vitro Antiglycation and Methylglyoxal Trapping Effect of Peppermint Leaf (*Mentha × piperita* L.) and Its Polyphenols

**DOI:** 10.3390/molecules28062865

**Published:** 2023-03-22

**Authors:** Izabela Fecka, Katarzyna Bednarska, Adam Kowalczyk

**Affiliations:** 1 Department of Pharmacognosy and Herbal Medicines, Faculty of Pharmacy, Wroclaw Medical University, ul. Borowska 211, 50-556 Wroclaw, Poland; 2 Committee for Therapeutics and Drug Sciences, Polish Academy of Sciences, pl. Defilad 1, 00-901 Warszawa, Poland

**Keywords:** *Mentha piperita*, polyphenols, flavonoids, eriocitrin, luteolin glycosides, glycation inhibitors, methylglyoxal, MGO trapping

## Abstract

The most significant reactive α-dicarbonyl RCS involved in the pathomechanism of glycation and related diseases is methylglyoxal (MGO). Hyperglycemia promotes the generation of MGO and leads to the formation of advanced glycation end products (AGEs). Therefore, MGO trapping and glycation inhibition appear to be important therapeutic targets in prediabetes, diabetes, and in the early prevention of hyperglycemic complications. Peppermint leaf is commonly used as herbal tea, rich in polyphenols. Eriocitrin, its predominant component, in a double-blind, randomized controlled study reversed the prediabetic condition in patients. However, the antiglycation activity of this plant material and its polyphenols has not been characterized to date. Therefore, the aim of this study was to evaluate the ability of a peppermint leaf dry extract and its polyphenols to inhibit non-enzymatic protein glycation in a model with bovine serum albumin (BSA) and MGO as a glycation agent. Peppermint polyphenols were also evaluated for their potential to trap MGO in vitro, and the resulting adducts were analyzed by UHPLC-ESI-MS. To relate chemical composition to glycation inhibitory activity, the obtained peppermint extract was subjected to qualitative and quantitative analysis. The capability of peppermint leaf polyphenols to inhibit glycation (27.3–77.2%) and form adducts with MGO was confirmed. In the case of flavone aglycones, mono- and di-adducts with MGO were observed, while eriodictyol and eriocitrin effectively produced only mono-adducts. Rosmarinic acid and luteolin-7-*O*-glycosides did not reveal this action. IC_50_ of the peppermint leaf dry extract was calculated at 2 mg/mL, equivalent to a concentration of 1.8 μM/mL of polyphenols, including ~1.4 μM/mL of flavonoids and ~0.4 μM/mL of phenolic acids. The contribution of the four major components to the anti-AGE activity of the extract was estimated at 86%, including eriocitrin 35.4%, rosmarinic acid 25.6%, luteolin-7-*O*-rutinoside 16.9%, luteolin-7-*O*-β-glucuronoside 8.1%, and others 14%. The effect of peppermint dry extract and polyphenols in inhibiting MGO-induced glycation in vitro was comparable to that of metformin used as a positive control.

## 1. Introduction

Non-enzymatic glycation is a reaction between the carbonyl groups of reducing sugars or their derivatives such as reactive carbonyl species (RCS) and the amino, guanidino, or thiol groups of some biomolecules including peptides, proteins, lipoproteins, and nucleic acids. Several physiological products of sugar autooxidation are known, as well as intermediates from glucose and fructose metabolism characterized by the presence of two adjacent carbonyl groups (α-dicarbonyl compounds). These compounds are ketoaldehydes or dialdehydes and are currently called RCS due to their high reactivity. The most important ketoaldehyde involved in the pathomechanism of non-enzymatic glycation and related diseases is methylglyoxal (MGO). Long-term hyperglycemia promotes the production of MGO, and this significantly increases glycation, leading, among other things, to the formation of irreversible advanced glycation end products (AGEs). AGEs can bind to specific receptors (RAGE) in the cell membrane and consequently trigger the nuclear factor κB (NF-κB) signaling pathway, which induces inflammation and oxidative stress and causes cell damage. In patients with diabetes mellitus, blood proteins such as hemoglobin and serum albumin are particularly vulnerable to glycation. MGO-derived AGEs have been shown to play a pivotal role in the onset and progression of vascular complications of diabetics. Methylglyoxal thus appears to be a significant contributor to endothelial dysfunction by increasing oxidation, as well as inducing inflammation and apoptosis [1,2,3,4]. Therefore, MGO scavenging and glycation inhibition are now recognized as promising therapeutic targets in diabetes, pre-diabetes, and early prevention of hyperglycemic complications.

Several substances of natural origin that can inhibit non-enzymatic protein glycation in the BSA-methylglyoxal and BSA-glucose systems are known. Such potential has been described, among others, for the polyphenols of rooibos, green and black tea [5,6]. Peppermint leaf is commonly used as herbal tea, rich in polyphenolic compounds. Its extracts have been shown not only to have a hypoglycemic effect but also to alleviate the symptoms of metabolic disorders in animal models [7,8,9]. Similar results were observed in a study involving young, healthy volunteers, in which peppermint leaf improved biochemical and anthropometric parameters that are cardiometabolic risk factors [10]. Additionally, eriocitrin, the major component of peppermint extracts, in a double-blind, randomized controlled study reversed the prediabetic condition in patients [11,12].

Peppermint (*Mentha × piperita* L.) is a hybrid of spearmint (*Mentha spicata* L.) and water mint (*Mentha aquatica* L.), and its leaves are used for both food and medicinal purposes [8,13]. The chemical composition of peppermint leaf is variable and depends on the geographic region from which it comes and the conditions of cultivation and storage. However, two main groups of active compounds can be differentiated—a volatile fraction including essential oil and a non-volatile fraction characterized by polyphenolic compounds [13,14,15,16,17]. The European Pharmacopoeia monograph for peppermint leaf dry extract (lat. *Menthae piperitae folii extractum siccum*) requires that it should contain not less than 0.5% rosmarinic acid [16]. Furthermore, the non-volatile fraction contains eriocitrin (eriodictyol-7-*O*-rutinoside) as the predominant polyphenolic metabolite, other glycosides of flavanone and flavone, as well as oligomeric caffeic acid esters [14,15,17].

The diverse chemical composition of *M. piperita* determines its biological and therapeutic indications. The European Medicines Agency’s Committee on Herbal Medicinal Products (EMA/HMPC) states that peppermint leaf is a traditional herbal medicine used for the symptomatic relief of digestive disorders such as indigestion and flatulence [17,18]. Medicines containing peppermint leaf preparation are usually used in liquid or solid form. In addition to the above-mentioned EMA/HMPC recommendations, compounds in peppermint leaf also display antioxidant, anti-allergic, anti-inflammatory, nociceptive, antimicrobial, antiviral, spasmolytic, choleretic, chemopreventive, hepatoprotective, and renoprotective effects [17]. However, the anti-glycation activity of this plant material and its polyphenolic components has not been characterized to date.

Therefore, the aim of our study was to evaluate the ability to inhibit the process of non-enzymatic protein glycation by a peppermint leaf dry extract and its major polyphenolic components in a model with bovine serum albumin and methylglyoxal as a glycation agent. Peppermint polyphenols were also evaluated for their potential to capture MGO in vitro, and the resulting adducts were analyzed by UHPLC-ESI-MS. The effect of individual extract components on the observed antiglycation properties was also investigated.

## 2. Results and Discussion

Peppermint leaf is a popular non-caffeine alternative to green or black tea. It is also used therapeutically for common digestive ailments. Current research indicates that it may also be effective in certain metabolic disorders. There is evidence from an animal model supporting its potential to lower blood levels of glucose, triacylglycerols, cholesterol and LDL (low-density lipoprotein) [7,8,9]. The effect of peppermint leaf on the biochemical and anthropometric profile of healthy volunteers was studied by Barbalho et al. [10]. Their results confirmed the ability of this plant material to reduce glycemia, triacylglycerols, total cholesterol, LDL-c, GOT (glutamic-oxaloacetic transaminase) and GPT (glutamic-pyruvic transaminase), and to increase HDL-c (high-density lipoprotein cholesterol). They also noted a reduction in blood pressure and body mass index (BMI). Thus, regular consumption of peppermint leaf may be beneficial for cardiometabolic health.

Among the pharmacologically active components of *M. piperita* are essential oil (monoterpenes) and polyphenols (flavonoids and phenolic acids). Menthol and menthone give peppermint preparations a specific refreshing taste. The constituents of the essential oil also exert a cholagogic effect, stimulating digestion and eliminating flatulence. However, in aqueous extracts, such as infusion and dry water extract, polyphenols are dominant and can play a pivotal role in the activity. The polyphenol profile of peppermint leaf, its water infusion and alcoholic tincture have been reported in some detail [14,15,18]. Their principal polyphenol is eriocitrin, accompanied by smaller amounts of glycosides of luteolin, apigenin, diosmetin, naringenin, eriodictyol and hesperetin. Another group comprises oligomeric caffeic acid esters such as rosmarinic and lithospermic acids, which are classified as phenolic acids. These compounds are known for their antioxidant properties [19,20]. Anti-glycation properties of some flavonoids and rosmarinic acid have also been confirmed [21,22,23,24]. Nevertheless, most flavanone and flavone glycosides have not been studied for this purpose.

The antioxidant potential of peppermint leaf was established using various methods. These properties included iron (III) reduction, iron (II) chelation, DPPH radical scavenging, and the ability to inhibit phospholipid peroxidation catalyzed by iron (III)-ascorbate [17,19,20]. In addition, there is clinical evidence supporting the beneficial effects of eriocitrin on the health of prediabetic patients [11,12]. Therefore, we decided to investigate the potential of peppermint leaf extract and its individual polyphenols to inhibit glycation and MGO uptake. Metformin, a standard oral hypoglycemic and antidiabetic agent was used as the reference substance (positive control).

### 2.1. Polyphenolic Profile of Peppermint Leaf Dry Extract

**Figure 1:**
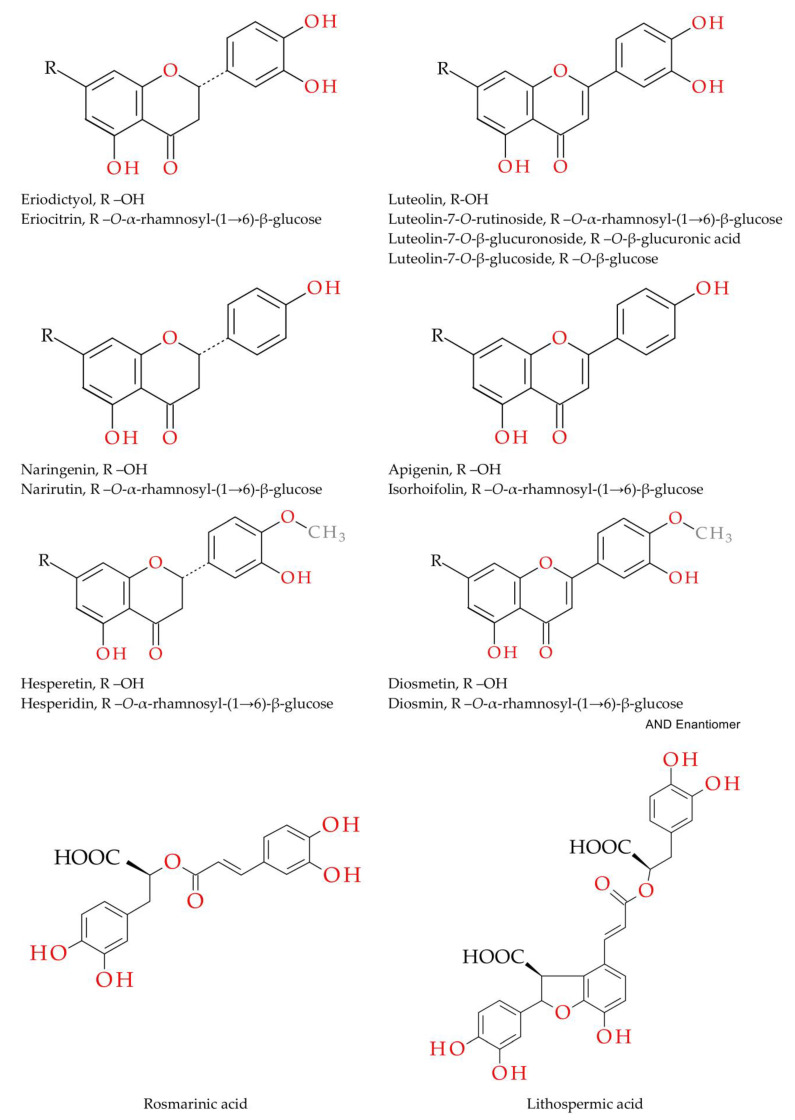
Structures of peppermint polyphenols.

In the present study, a dry extract was obtained from a water infusion of peppermint leaf using the solid-phase extraction (SPE) method. SPE is one of the simplest yet most efficient and versatile techniques for concentrating samples and extracts. With properly selected parameters, it also allows partial purification of the concentrated material from substances of high polarity such as salts and sugars. The peppermint leaf dry extract, containing partially purified polyphenols, was subjected to compositional analysis by UHPLC-ESI-MS against authentic flavonoid and phenolic acid standards. The qualitative composition of polyphenols is shown in Table 1. It lists only the components that occurred at relatively high levels. The chemical composition of the prepared extract did not generally differ from other *M. piperita* preparations [14,15]. The chemical structures of the main identified compounds are in Figure 1.

**Figure 2:**
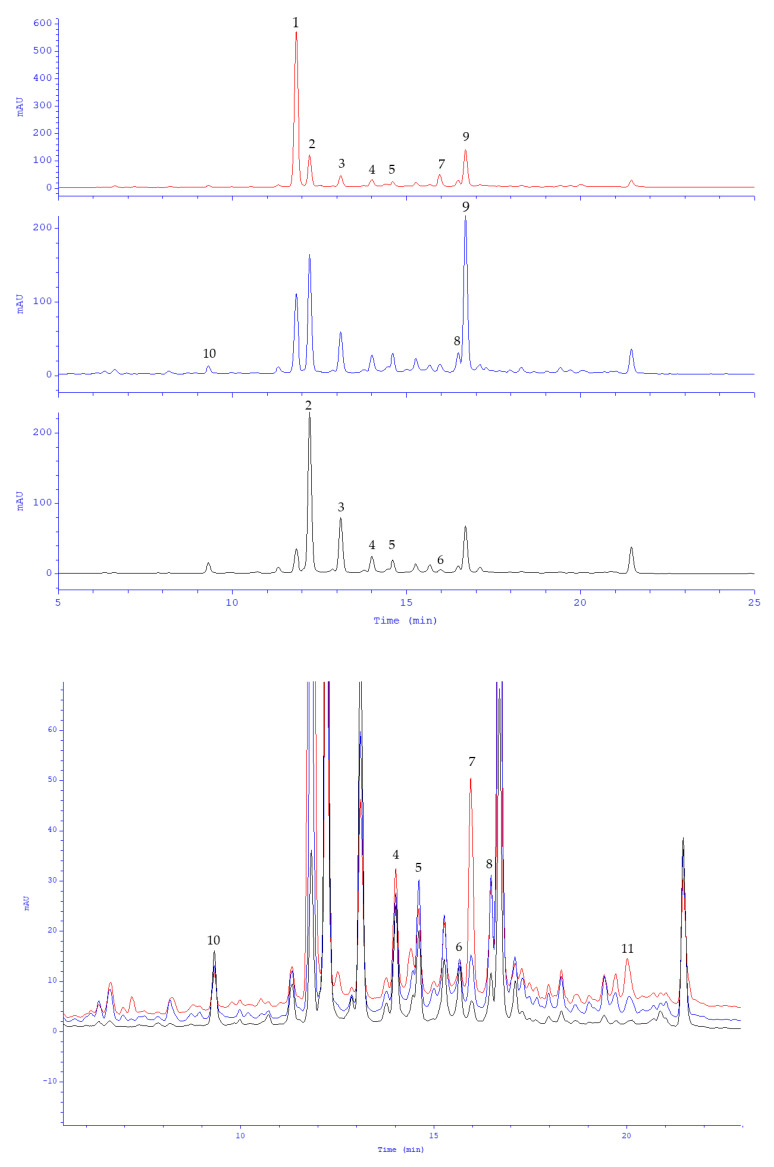
HPLC chromatograms of peppermint leaf dry extract (1.5 mg/mL, 50% aq. methanol); red at 280 nm, blue 320 nm, and black 360 nm. Peak labeling: 1, eriocitrin; 2, luteolin-7-*O*-rutinoside; 3, luteolin-7-*O*-β-glucuronoside; 4, narirutin; 5, isorhoifolin; 6, diosmin; 7, hesperidin; 8, lithospermic acid; 9, rosmarinic acid; 10, caffeic acid; 11, eriodictyol.

The content of the major components of the extract was determined using the HPLC-DAD method previously developed and validated for plant materials in the Lamiaceae family. This method has been used successfully in several studies [14,15]. A typical HPLC-DAD chromatogram of peppermint leaf dry extract (1.5 mg/mL, solution in 50% aq. methanol) is provided in Figure 2. As expected, the dominant compound was eriodictyol-7-*O*-rutinoside, known as eriocitrin (285.4 mg/g = 478.4 μM/g). Luteolin-7-*O*-rutinoside (syn. scolymoside, 78.5 mg/g = 132.1 μM/g) and luteolin-7-*O*-β-glucuronoside (27.6 mg/g = 59.6 μM/g) were reported in substantially lower amounts (Table 2). These were followed by rosmarinic acid (57.8 mg/g = 160.5 μM/g), hesperetin-7-*O*-rutinoside (syn. hesperidin, 22.9 mg/g = 37.4 μM/g), lithospermic acid (8.3 mg/g = 15.4 μM/g), diosmetin-7-*O*-rutinoside (syn. diosmin, 4.7 mg/g = 7.8 μM/g), apigenin-7-*O*-rutinoside (syn. isorhoifolin, 3.4 mg/g = 5.8 μM/g) and naringenin-7-*O*-rutinoside (syn. narirutin, 1.2 mg/g = 2.1 μM/g), as well as luteolin-7-*O*-β-glucoside, eriodictyol, luteolin, caffeic acid, and others (below 1 mg/g). Flavanone and flavone 7-*O*-rutinosides were predominant components (396.1 mg/g = 663.7 μM/g). The aglycones accompanied their own glycosides in negligible amounts. There were a total of 491 mg (904 μM) polyphenols in 1 g of the dry extract, including flavonoids 424.4 mg (726 μM) and phenolic acids 66.6 mg (179 μM). More than half of the polyphenolic components consisted of eriocitrin (~53%), with smaller percentages noted for luteolin glycosides and rosmarinic acid (~21% and 18%, respectively). The four major *M. piperita* compounds accounted for up to 92% of the polyphenol sum. The prepared dry extract of peppermint leaf met the requirements of the European Pharmacopoeia monograph for a minimum content of rosmarinic acid.

### 2.2. Anti-Glycation Activity of Peppermint Leaf Dry Extract and Its Polyphenolic Components

Excessive consumption of simple sugars, glucose and fructose, is one of the reasons for the epidemic of obesity, type 2 diabetes, metabolic syndrome, and cardiovascular disease. Non-enzymatic and enzymatic oxidation of monosaccharides leads to excessive production of RCS, carbonyl stress and related oxidative stress. The consequence is inflammation and malfunction of many tissues and organs, including the liver, kidneys, blood vessels and nervous tissue. These disorders mutually reinforce each other and the accompanying pathologies accumulate with increasing age, which is associated with the progression of cardiometabolic factors such as insulin resistance, impaired glucose tolerance, hyperglycemia, hyperlipidemia, hypercholesterolemia, hypertension and central adiposity [1,2,3,4,25,26,27,28,29,30,31,32,33,34,35]. Belonging to the physiological α-ketoaldehydes, methylglyoxal is a highly reactive metabolite known for its harmful effects. It interacts with the arginine, lysine, and cysteine residues of peptides, proteins, and lipoproteins, causing their post-translational modification, and leading to impaired function of many enzymes, peptide hormones, transport, defense, membrane, structural proteins, and others. MGO-derived modifications also facilitate the formation of cross-links of proteins that alter their higher-order structure and conformation. The process of glycation is long-term and proceeds in several steps. Initially, reversible N-substituted imines (Schiff bases) are formed, which are then converted into more stable irreversible ketoamines (Amadori products). These products undergo further conversions, including dehydration, oxidation and condensation. The resulting final compounds are called advanced glycation end products. AGEs accumulate especially on long-lived proteins such as serum albumin, collagen, lens α-crystallin and hemoglobin (Hb) [28,29,30,31,32]. Increased levels of MGO-derived AGEs have been found in the serum of diabetic patients. In addition, MGO concentrations in blood samples from patients with diabetes correlate positively with the duration of the disease. In laboratory tests, incubation of Hb with MGO resulted in the formation of hydroimidazolone derivatives on arginine residues. The dominant arginine adduct was reported to be N^δ^-(5-hydro-5-methyl-4-imidazolon-2-yl)-ornithine (MG-H1). The sites of modification were recognized to be arginine residue 31 in the α-chain, as well as arginine residues 30, 40, and 104 in the β-chain. Hb-MGO adducts presumably may play a critical role in inducing vascular endothelial cell injury [31]. An insulin-MGO adduct was also identified. Methylglyoxal modifies insulin by attaching to arginine residues at position 22 of the B chain. This insulin-MGO adduct reduces in vitro insulin-mediated glucose uptake, impairs autocrine control of insulin secretion, and decreases insulin clearance, leading to insulin resistance [33]. Serum albumin is also modified by MGO. After one day of incubation with MGO, 21 of the 23 arginine residues were irreversibly modified in BSA. In the same conditions, 19 of the 24 arginine residues were modified in human serum albumin (HSA). Likewise, many of the lysine residues in BSA and HSA have fused with MGO (23 of 59 and 11 of 59, respectively). MGO-modified albumin loses its antioxidant capacity and has reduced binding activity for some physiologically relevant compounds and drugs [34]. For these reasons, among others, AGEs, including MG-H1, are now considered risk factors not only for diabetic complications, but also for lifestyle-related diseases. Clinical conditions that can be induced by AGEs are micro- and macroangiopathies such as retinopathy, nephropathy, neuropathy, arterial stiffness, arteriosclerosis, cardiovascular disease, as well as hepatic steatosis, joint stiffness, senile cataracts and Alzheimer’s disease [1,2,3,4,32,35].

**Figure 3:**
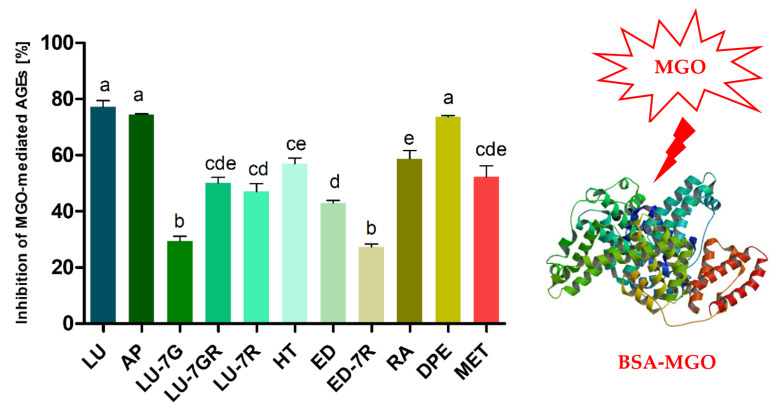
Antiglycation activity after seven-day incubation of bovine serum albumin with methylglyoxal (0.5 mM) and the test compound (1.5 mM) or peppermint leaf dry extract (3 mg/mL) expressed as % inhibition of MGO-induced AGE formation. Results are representative of three experiments performed as mean ± SD. Data were analyzed by one-way ANOVA (*p* < 0.0001) followed by Tukey’s multiple comparisons test; values not sharing a common letter are significantly different. Abbreviations: AP, apigenin; LU, luteolin; LU-7GR, luteolin-7-*O*-β-glucuronoside; LU-7R, luteolin-7-*O*-rutinoside; LU-7G, luteolin-7-*O*-β-glucoside; HT, hesperetin; ED, eriodictyol; ED-7R, eriocitrin; RA, rosmarinic acid; DPE, peppermint leaf dry extract; MET, metformin.

Along with HSA, BSA is the most commonly used in model studies of compounds with antiglycation, anti-AGE and anti-RCS potential. Figure 3 summarizes the results of the glycation inhibition test in the BSA-methylglyoxal model obtained in our study. Peppermint polyphenols and the extract were examined at a concentration of 1–1.5 μM/mL and 1.5–3 mg/mL, respectively. The greatest anti-AGE and anti-MGO effect was noted for luteolin (77.2 ± 7.8%) followed by apigenin (74.5 ± 0.6) and peppermint leaf dry extract (73.7 ± 1.3%). Nevertheless, these differences were not statistically significant. Rosmarinic acid (58.7 ± 10.2%), hesperetin (56.9 ± 4.4%), luteolin-7-*O*-β-glucuronoside (50.1 ± 7.1%), luteolin-7-*O*-rutinoside (47.0 ± 9.8%) and eriodictyol (43.0 ± 2.6%) showed an intermediate action. The statistically significant weakest antiglycation effects were observed for luteolin-7-*O*-β-glucoside (29.3 ± 6.1%) and eriocitrin (27.3 ± 3.9%). Under analogous conditions, the antidiabetic metformin inhibited glycation by 52.3% ± 13.8%. The complete dry extract of peppermint leaf at a concentration of 3 mg/mL showed statistically significantly larger effects than each of its individual components, with the exception of flavone aglycones. However, aglycones were noted in the extract in very low amounts.

IC_50_ of the peppermint leaf dry extract was calculated at 2 mg/mL, equivalent to a concentration of 1.8 μM/mL of polyphenols, including ~1.4 μM/mL of flavonoids and ~0.4 μM/mL of phenolic acids. Eriocitrin accounted for 1 μM/mL, luteolin glycosides 0.4 μM/mL, rosmarinic acid 0.3 μM/mL, and others were approximately 0.1 μM/mL. In the same conditions, the IC_50_ values of eriocitrin, luteolin-7-*O*-rutinoside, luteolin-7-*O*-β-glucuronoside and rosmarinic acid were found to be 2.7, 1.6, 1.5 and 1.3 μM/mL. For metformin, it was 1.4 μM/mL. The contribution of the four major components to the antiglycation activity of the peppermint leaf dry extract was estimated at 86% (based on concentration in μM/mL and % inhibition calculated from regression equations), including eriocitrin 35.4%, rosmarinic acid 25.6%, luteolin-7-*O*-rutinoside 16.9%, luteolin-7-*O*-glucuronoside 8.1%, and others 14%. The above analyses indicate that the principal component of the extract with antiglycation activity is eriocitrin. The contribution of rosmarinic acid and luteolin glycosides was at a similar but lower level (~25%). The additive effect of MGO trapping for a mixture of the flavonoids quercetin and phloretin was described by Shao et al. [21]. It is likely that a similar phenomenon occurs for peppermint leaf polyphenols. Flavonoid rutinosides, unfortunately, show much lower bioavailability than simple glycosides or flavonoid aglycones. However, it should be noted that the activity of aglycones released by intestinal biotransformation is much higher. Moreover, some of their human metabolites, e.g., 7-*O*-, 3′-*O* and 4′-*O*-glucuronosides of luteolin (syn. glucuronides) [36,37], may retain at least part of the activity. On the other hand, phenolic acids such as rosmarinic acid have a slightly higher bioavailability [38]. The effect of peppermint leaf polyphenols on glycation and MGO levels in the intestinal lumen and potential interactions with the microbiota should also be taken into consideration.

The anti-AGE effect of polyphenols is related to their protective action against chemical modifications of substrates involved in the glycation reaction, such as binding to groups involved in the initiation of glycation or inhibiting the oxidation of simple sugars and ketoamines (Amadori products). A key component of this effect is the ability to capture glycation- or oxidation-initiating factors (RCS, ROS) [21,22,23,39]. Anti-MGO potential of luteolin-7-*O*-rutinoside, luteolin-7-*O*-β-glucuronoside, luteolin-7-*O*-β-glucoside and eriodictyol-7-*O*-rutinosides with regard to aglycones was lower, at 35–62% of their activity. This loss of effectiveness in inhibiting glycation shown by 7-*O*-substituted flavones and flavanones may be related to the partial or complete abolition of MGO-trapping capacity. Therefore, in another experiment, we tested the ability of peppermint polyphenols and extract to capture MGO.

### 2.3. MGO-Trapping Potential of Peppermint Leaf Polyphenols

Several synthetic medicines and plant polyphenols are known to have the capacity for MGO scavenging [5,39,40,41]. However, only metformin is of practical therapeutic use in patients with type 2 diabetes and insulin resistance. The products of nucleophilic addition to the carbonyl group of methylglyoxal are compounds with attached MGO in the form of a side chain and/or heterocyclic ring. Using metformin as an example, these include imidazolinone and triazepinone derivatives [41]. Research by Bhuiyan et al. [42] on quercetin-MGO adducts shows that they can have a hemiacetal or hemiketal structure and feature a dihydrofuran ring fused to the flavonoid benzene ring. The presence of these new compounds in the reaction mixture is confirmed using chromatographic methods coupled to a mass detector (MS). Mono- and di-adducts of MGO with polyphenols are characterized by a pseudomolecular ion with an exact mass of 72.02 Da and 144.04 Da higher than the precursor ion, respectively [21,39,40,42].

With the help of UHPLC-ESI-MS, we examined the ability of *M. piperita* polyphenols to form adducts with MGO. The results from the trapping test are summarized in Table 3. An analogous set of peppermint polyphenols was used as in the glycation inhibition assay. The MGO-trapping ability of other flavones and flavanones such as vitexin (apigenin-8-*C*-β-glucoside), isovitexin (apigenin-8-*C*-β-glucoside), hesperidin, hesperetin, diosmin and diosmetin have been reported by our group previously [5,39]. Mono-adducts with MGO have been noted for all flavonoid aglycones, both flavones and flavanones. Apigenin, luteolin, and hesperetin each provided two mono-MGO isomers. Di-MGO adducts were also formed by reaction with luteolin, apigenin and hesperetin (one each). The results obtained for these compounds were consistent with the literature data [39,43]. In our previous study for hesperetin and diosmetin three MGO adducts each were observed, two mono-MGO and one di-MGO [39]. Their 7-*O*-rutinosides, however, showed different properties. Diosmin did not trap MGO under the test conditions, while hesperidin was the source of six isomeric mono-MGO adducts. Flavanones contain a chiral C-2 atom and can therefore exist as (*2S*)- and (*2R*)-enantiomers, e.g., in citrus (*2S*)-hesperidin is the main one. For eriodictyol and eriocitrin, which also occur in (*2S*)- and (*2R*)-configurations, we observed four mono-MGO adducts each, with pseudomolecular ions at *m*/*z* 359.08 Da and 667.19 Da, respectively. Among the mono-MGO-eriocitrins, isomer 3 (21.40 min, *m/z* 667.1873 Da) was the predominant one. Unexpectedly, luteolin-7-*O*-glycosides (rutinoside, glucuronoside and glucoside) did not show the ability to capture methylglyoxal, probably due to glycosylation of the hydroxyl group at the C-7 position of the benzene ring. No adducts with rosmarinic acid were confirmed either. Former studies have revealed that the phloroglucinol arrangement in the flavonoid framework is an essential element for MGO trapping [5,21,44]. Phloroglucinol tested under the same conditions inhibited by 60% the production of MGO-induced AGEs with the formation of mono-, di- and tri-MGO adducts [5]. It is likely that for some polyphenols, the antiglycation effect is solely related to their antioxidant and transition metal ion chelating activity [23,24]. They may act as radical scavengers and/or metal chelators. The presence of reactive oxygen species (ROS) during the glycation process with trace amounts of metal ions has been proven. However, metal chelation seems to be less involved in the anti-AGE activity [45]. Polyphenols can also form complexes with serum albumin and block access to the glycation site. Interactions of BSA and HSA with polyphenols, including eriocitrin, luteolin and rosmarinic acid, were analyzed by spectroscopic and molecular docking methods [46,47,48]. Phenolic compounds induced conformational changes in the albumin protein, and the main forces involved in binding were hydrophobic interactions.

To sum up, (1) non-methylated flavones demonstrated substantially higher antiglycation and MGO-trapping potential than non-methylated flavanones. The difference in anti-MGO activity of luteolin and eriodictyol, as well as luteolin-7-*O*-rutinoside and eriocitrin, was 44% and 42%, respectively. Generally, the double bond between C-2 and C-3 enhances anti-AGE efficacy. (2) Glycosylation of the 7-hydroxyl group of flavones and flavanones substantially reduced activity (e.g., luteolin vs. luteolin-7-*O*-rutinoside by 39%, eriodictyol vs. eriocitrin by 37%, hesperetin vs. hesperidin by 9%). With regard to flavone 7-*O*-glycosides, there was complete abolition of MGO-trapping ability (7-*O*-β-glucoside, 7-*O*-β-glucuronoside and 7-*O*-rutinoside of luteolin, as well as diosmin). Identical results were obtained by Li et al. [49] in the BSA-glucose model for hesperetin, hesperidin, and hesperetin-7-*O*-glucoside (1 mM; the inhibitory rate was 56.7%, 45.8% and 43.5%, respectively), as well as their enantiomers (*2S*, *2R*). Interesting features were noted before for flavone *C*-glycosides (vitexin and isovitexin), which, despite glycosylation of one of the two positions important in the addition reaction (C-8 or C-6), retained the ability to take up MGO with the formation of two isomeric mono-MGO adducts [5]. It also turned out that (3) the methylation of hydroxyl groups in C-4′ of the phenyl ring of flavanones may alter their anti-MGO properties. The methylated counterpart captured two MGO molecules and more strongly inhibited MGO-induced glycation (hesperetin vs. eriodictyol by 24%). This effect may be related to a change in the stability of the flavanone heterocyclic ring (e.g., methoxyl at C-4′ is less prone to oxidation and semiquinone formation). A different outcome was observed with 4′-methoxyflavones; their potential against analogous derivatives of 4′-methoxyflavanones decreased (diosmetin vs. hesperetin by 45%, and diosmin vs. hesperidin by 20%) [40]. Similar conclusions were reached by Shao et al. [21] in a study on the potential of flavonoids to scavenge MGO, as well as by Matsuda et al. [45] from results obtained in the BSA-glucose model.

A trapping test conducted on peppermint leaf extract confirmed the observations gained for its individual compounds. Since the predominant component of *M. piperita* was eriocitrin, and 7-*O*-glycosides of luteolin did not capture MGO, the main signals in the extract were four pseudomolecular ions derived from mono-MGO-eriodictyol isomers (Table 3).

**Figure 4:**
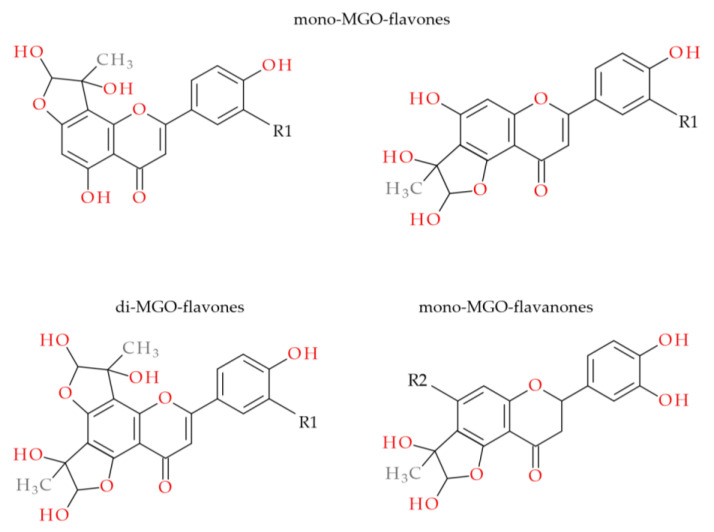
Proposed hemiacetal structures of mono-MGO and di-MGO adducts of luteolin (R1, -OH), apigenin (R1, -H), eriodictyol (R2, -OH) and eriocitrin (R2, -O-rutinoside).

**Figure 5:**
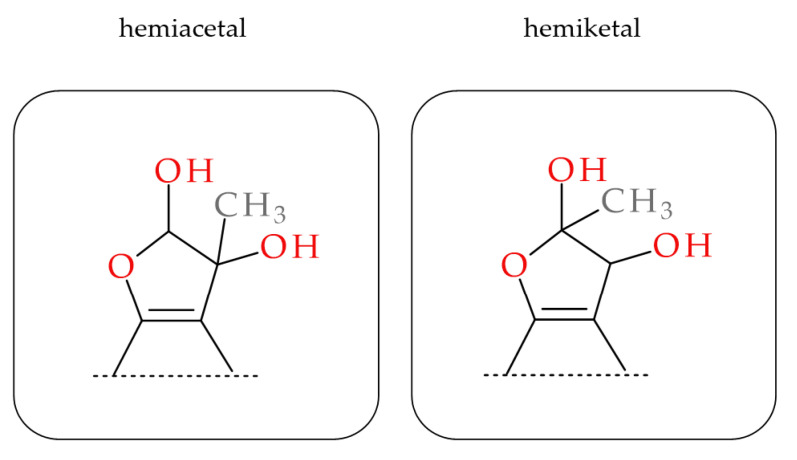
Scheme of the hemiacetal and hemiketal adducts of MGO with flavones and flavanones (2,3-dihydrofuran ring).

**Figure 6:**
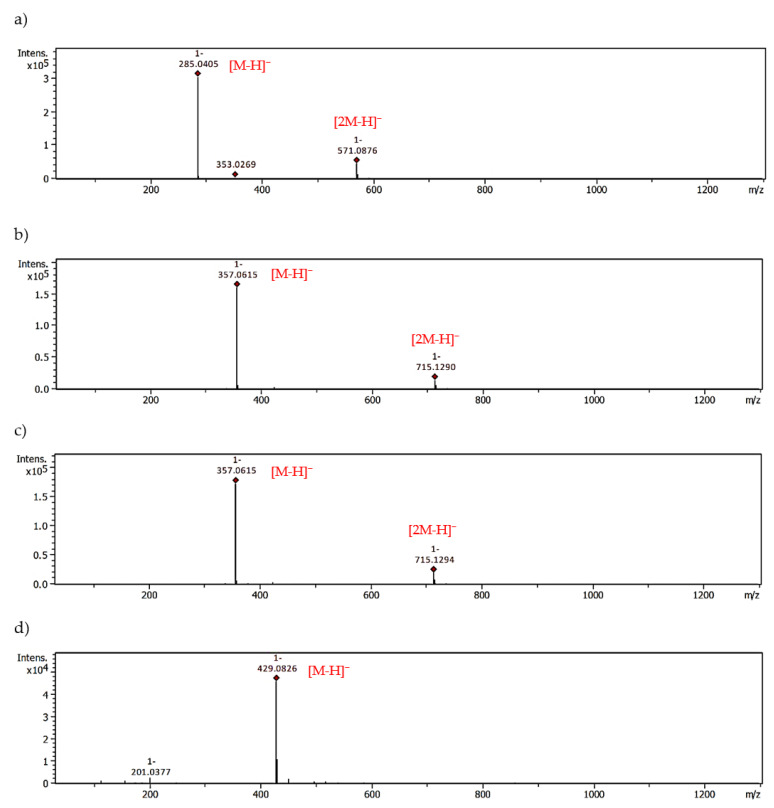
MS spectra of luteolin and luteolin-MGO; (**a**) authentic standard, (**b**) mono-adduct 1, (**c**) mono-adduct 2, (**d**) di-adduct with MGO.

**Figure 7:**
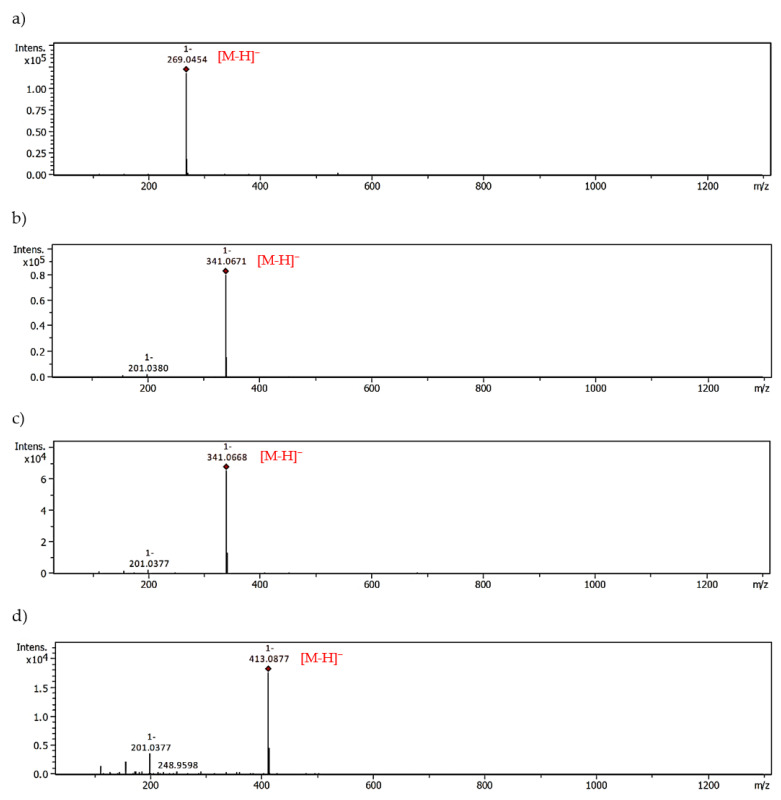
MS spectra of apigenin and apigenin-MGO; (**a**) authentic standard, (**b**) mono-adduct 1, (**c**) mono-adduct 2, (**d**) di-adduct with MGO.

**Figure 8:**
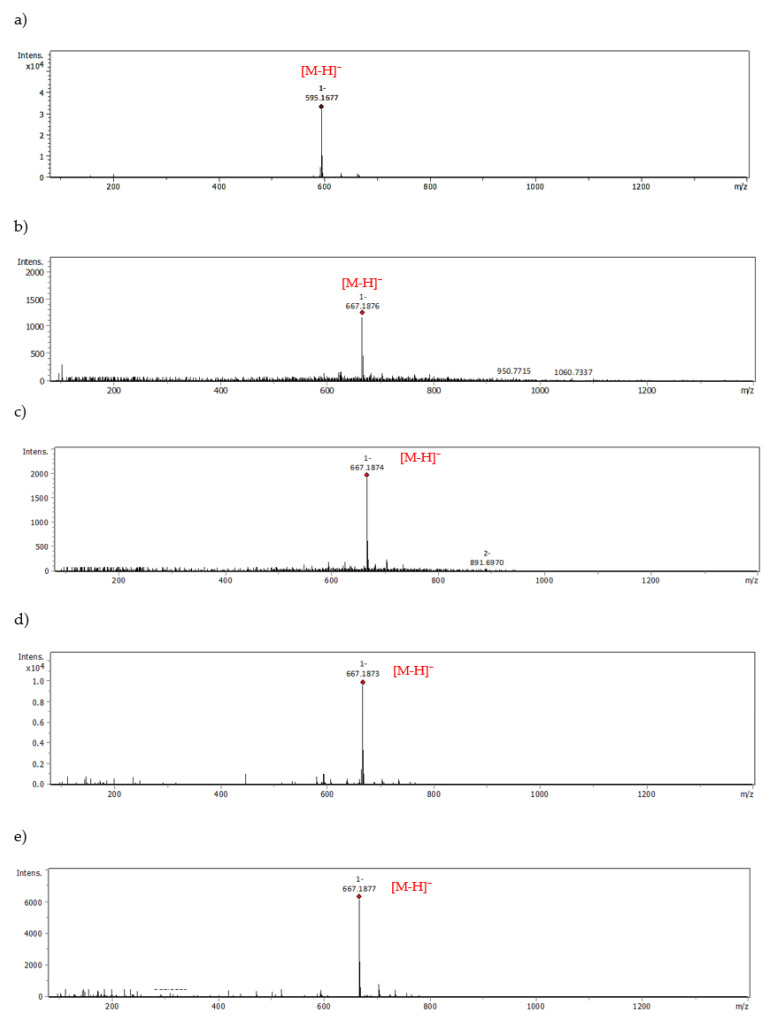
MS spectra of eriocitrin and eriocitrin-MGO; (**a**) authentic standard, (**b**) mono-adduct 1, (**c**) mono-adduct 2, (**d**) mono-adduct 3, (**e**) mono-adduct 4.

The possible heterocyclic structures of the mono- and di-methylglyoxal adducts of luteolin, apigenin, eriodictyol and eriocitrin are shown in Figure 4. Nucleophilic addition can result in the formation of isomers that differ in the site of MGO attachment in the benzene ring of the flavonoid (at the C-6 or C-8 position next to the free -OH groups involved in the reactions), as well as in the arrangement and spatial orientation of the functional groups (-OH, -CH_3_) in the newly formed 2,3-dihydrofuran heterocyclic ring. Depending on which MGO carbonyl (aldehyde or ketone) participates in the reaction, the adduct can be either a hemiacetal or a hemiketal. According to a structural study of quercetin-MGO adducts by Bhuiyan et al. [42], hemiacetal is most likely the dominant form. Figure 5 summarizes the schematic 2,3-dihydrofuran structures of hemiacetal and hemiketal. The subsequent Figure 6, Figure 7 and Figure 8 show the pseudomolecular ions of the mono-MGO and di-MGO adducts of luteolin, apigenin, and eriocitrin. Figures S1–S13 in supplementary data present MS spectra of luteolin, apigenin, and eriocitrin authentic standards and their MGO adducts.

Eriocitrin is a 7-*O*-rutinoside of eriodictyol with a health-promoting effect, found mainly in peppermint leaves, and lemon and lime fruits [13,14,15,50]. It has been shown to have antioxidant, anti-inflammatory, lipid-lowering, hypoglycemic and nephroprotective action [11,12,19,51,52,53,54,55,56,57,58]. Eriocitrin reduces oxidative stress and systemic inflammation and improves lipid and glucose metabolism [11,12,51,52,53,54,55,56,57]. It also ameliorates diet-induced hepatic steatosis by activating mitochondrial biogenesis [58]. In our study, we have demonstrated its anti-MGO and anti-AGE effect. These broad biological properties of eriocitrin are of great interest, and the revealed mechanisms of its action may become a starting point for further in vivo experiments.

## 3. Materials and Methods

### 3.1. Chemicals

Methanol, acetonitrile (LC gradient grade and LC-MS grade), water (LC-MS grade), DMSO, 98–100% formic acid, methylglyoxal (MGO, 40% in water), and bovine serum albumin (BSA) were purchased from Merck–Sigma–Aldrich (Poland–Sigma-Aldrich, Poznań, Poland). Glacial acetic acid, NaCl, KCl, Na_2_HPO_4_, and KH_2_PO_4_ (reagent grade) were obtained from Chempur (Piekary Śląskie, Poland). All other reagnts, unless stated otherwise, were of analytical grade and purchased from Chempur. Water was glass-distilled and deionized.

### 3.2. Authentic Standards

Eriodictyol (CAS No. 552-58-9), luteolin (CAS No. 491-70-3), luteolin-7-*O*-β-glucoside (CAS No. 5373-11-5), apigenin-7-*O*-rutinoside (CAS No. 552-57-8, syn. isorhoifolin), naringenin-7-*O*-rutinoside (CAS No. 14259-46-2, syn. narirutin), hesperetin-7-*O*-rutinoside (CAS No. 520-26-3, syn. hesperidin), diosmetin-7-*O*-rutinoside (CAS No. 520-27-4, syn. diosmin), caffeic acid (CAS No. 331-39-5), and rosmarinic acid (CAS No. 20283-92-5) were purchased from Extrasynthese (Genay, France). Metformin hydrochloride (CAS No. 1115-70-4) was purchased from Merck-Sigma-Aldrich (Darmstadt, Germany). Eriocitrin (Cas No. 13463-28-0) and luteolin-7-*O*-rutinoside (CAS No. 20633-84-5, syn. scolymoside) were isolated from *M. piperita* leaf, as described previously [59]. Luteolin-7-*O*-β-glucuronoside (CAS No. 29741-10-4, syn. luteolin-7-*O*-β-glucuronide) and lithospermic acid (CAS No. 28831-65-4) were isolated from *Thymus serpyllum* L. herb [60].

Stock solutions (1 mg/mL) for quantitative analysis were made by dissolving 2–5 mg of flavonoid in 2–5 mL of methanol. Working standard solutions in the range of 10–250 g/mL (6 measurement points for each pattern) were prepared by mixing with 50% aq. methanol (*v*/*v*), filtered through hydrophilic Millex Syringe Filters, Durapore 0.45 μm and 0.22 μm (Merck-Sigma-Aldrich, Darmstadt, Germany) and stored at −20 °C.

### 3.3. Plant Material and Extract Preparation

*Mentha × piperita* L. leaves (*M. piperita*) were bought from the herbal company KAWON (Gostyń, Poland), GMP and ISO 9002 certified. Peppermint leaf was powdered (10 g) (IKA A11B analytical mill; IKA Warsaw, Poland), added to boiling distilled water (500 mL), stirred, and after 30 min filtered through filter paper (Whatman No. 1, Little Chalfont, Buckinghamshire, UK). The peppermint water infusion was concentrated using the solid-phase extraction (SPE) method. The filtrate (400 mL) was acidified with formic acid (2 mL) and adsorbed onto an octadecyl column (2 × 10 cm, BAKERBOND Octadecyl 40 m Prep LC Packing, J.T. Baker, Phillipsburg, NJ, USA). The column was dried under vacuum (30 min) and the polyphenols were eluted with methanol (3 × 100 mL). Eluates were combined and concentrated at 40 °C (Rotavapor R-300, BÜCHI Labortechnik AG, Flawil, Switzerland). The concentrated eluates were allowed to dry, yielding a peppermint leaf dry extract. The extraction was carried out according to a previously described method [59,60].

### 3.4. Chemical Composition of Peppermint Leaf Dry Extract

The presence of flavonoids and phenolic acids in peppermint leaf dry extract was confirmed by the UHPLC-ESI-MS method described previously by Bodalska et al. [15].

The content of the dominant compounds was determined by HPLC-DAD. This method was developed and optimized previously by Fecka and Turek [14]. Peppermint leaf dry extract was separated in a Smartline system (Knauer, Berlin, Germany) equipped with a pump (Managare 5000), a dynamic mixing chamber (V7119-1), a DAD 2800 detector, a manual 6-port 2-channel injection valve (A1366) and a column thermostat (Jetstream Plus). The separation was performed on a Hypersil GOLD C18 column (250 × 4.6 mm, particle size 5 µm) with a C18 precolumn (10 × 4.6 mm, size 5 µm) (Thermo Fisher Scientific, Waltham, MA, USA). The mobile phases were (*v*/*v*): 5% formic acid in water (solvent A), and 5% formic acid in acetonitrile (solvent B). The following gradient program was used: 10%→40%→70% B in A at time points of 0→25→30 min. Before each analysis, the column was washed with mobile phase B (10 min) and stabilized with eluent under initial conditions (5 min). The flow rate was 1.0 mL/min, the injection volume 20 μL. The thermostat was set at 20 °C. UV/Vis spectra were taken in the wavelength range of 200–600 nm, with steps of 2 nm. Flavanones were evaluated at 280 nm, flavones at 360 nm, and phenolic acids (caffeic acid derivatives) at 320 nm. Data were processed using EuroChrom for Windows Basic Edition V3.05 (V7568-5).

The HPLC-DAD method was validated according to ICH guidelines for linearity, detection and quantification limits, intra- and inter-day precision. Calibration curves for the quantified compounds were determined from 6 measurement points, and double injections were performed for each concentration. The correlation coefficients of the calibration curves (*r*) used in the calculations were above 0.999 [14,15].

The content of individual polyphenols (mg/g dry extract) was determined using the external standard method based on the area of the corresponding peaks. Polyphenol content was then converted to micromolar concentrations. The contents of flavonoids, phenolic acids, and polyphenols and their percentages in the polyphenol fraction (non-volatile compounds) were also calculated.

### 3.5. Antiglycation Non-Enzymatic Assay (BSA-Methylglyoxal Model)

For this purpose, 21.2 μM bovine serum albumin was incubated with 0.5 mM methylglyoxal and 1–1.5 mM of the test compound or peppermint leaf dry extract (1–3 mg/mL) in 100 mM PBS solution, pH 7.4, with 0.02% sodium azide. The reaction mixture was shaken (50 rpm) at 37 °C, for 7 days, in closed vials without light. The fluorescent intensity of MGO-mediated AGEs formed during incubation was analyzed using a Synergy HTX Multi-Mode Microplate Reader (BioTek Instruments Inc., Winooski, VT, USA) at a wavelength of 360 nm for excitation (λ_ex_) and 460 nm for emission (λ_em_). Data processing was performed using Gen5 Software (BioTek In-struments Inc., Winooski, VT, USA). Measurements from in vitro experiments were made in triplicate, and the percentage inhibition of AGE formation was calculated from the following equation:

%Inhibition of MGO-mediated AGEs = {1 − [(FI_1_)/(FI_0_)]} × 100

where FI_0_ is mean fluorescence intensity of the blank sample and FI_1_ is the mean fluorescence intensity of the tested sample [5].

The percentage contribution of each component to the activity of the peppermint leaf dry extract was calculated from regression equations for the relationship between the concentration [μM] and % inhibition. These were then related to the overall effect exerted by the extract.

### 3.6. MGO Trapping and Adduct Analysis

The direct MGO-trapping capacity of peppermint polyphenols was investigated according to the method of Shao et al. [21], with slight modifications. Briefly, 0.6 mM of freshly prepared MGO solution was incubated with 0.2 mM of an individual compound and 0.1 M PBS (pH 7.4) at 37 °C and shaken at 50 rpm for 1 h. The reaction was termined by adding 2.5 μL of glacial acetic acid and transferring samples to an ice water bath. Samples were then filtered through Millex hydrophilic syringe filters (Durapore 0.22 μm) and analyzed using UHPLC-ESI-MS (described below).

### 3.7. Analysis of the Polyphenol-MGO Adducts

Adducts of peppermint polyphenols with MGO were analyzed by UHPLC-ESI-MS using a Thermo Scientific Dionex UltiMate 3000 UHPLC system (Thermo Fisher Scientific; Waltham, MA, USA) incorporated with a Compact ESI-QTOF-MS (Bruker Daltonics; Bremen, Germany), a quaternary pump (LPG-3400D) and an UltiMate 3000 RS autosampler (WPS-3000). The reaction mixtures were separated on a Kinetex C18 column (150 × 2.1 mm, particle size 2.6 μm) (Phenomenex; Torrance, CA, USA) at 40 °C (a temperature-controlled column compartment, TCC-3000). The mobile phases were (*v*/*v*): 0.1% formic acid in water (solvent C), and 0.1% formic acid in acetonitrile (solvent D). The following gradient program was used: 3%→35%→80% D in C, at time points of 0→12→14 min. The system was then washed in mobile phase D (3 min) and stabilized with eluent under initial conditions before the next analysis (2 min). The flow rate was 0.3 mL/min, the injection volume 2.5 μL. Negative ion mode (ESI^−^) was used for data acquisition. Nitrogen was used as a nebulizing gas at 210 °C, 2.0 bar pressure, and flow 0.8 L/min. For internal calibration, sodium formate clusters (10 mM) were used. Additional operating conditions of the mass spectrometer were as follows: the capillary voltage was set at 5 kV, the collisional energy was 8.0 eV, and for the MS^2^ mode, it was 40 eV. Data were analyzed using Compass Data Analysis software (Bruker Daltonics; Bremen, Germany).

### 3.8. Statistical Analysis

All data are presented as mean ± standard deviation (SD). Data were analyzed using the Shapiro-Wilk test to assess the normality of distribution, followed by one-way analysis of variance (ANOVA) with Tukey’s multiple comparison test using the GraphPad Prism 9 software, and *p* values equal to or less than 0.05 were considered significant.

## 4. Conclusions

Reactive dicarbonyls, such as methylglyoxal, cause post-translational modifications of physiologically relevant bimoolecules, changing their structure and impairing function. The AGEs formed as a result of such activity then initiate many unfavorable processes associated with cardiometabolic disorders. The strategy of inhibiting glycation and removing MGO by capturing or preventing its formation appears to be a promising therapeutic direction. In this study, peppermint leaf dry extract and its polyphenols revealed the capability to inhibit MGO-induced glycation in vitro. Both flavonoids and rosmarinic acid showed significant antiglycation activity. However, only for flavones and flavanones was the potential to capture MGO confirmed. Among the compounds tested, luteolin and apigenin were the most active. Eriocitrin, the principal component of peppermint leaf, was less effective in inhibiting glycation. Nevertheless, due to its high content in the extract, its contribution to the effect was superior to other components. In addition, unlike 7-*O*-glycosides of luteolin, eriocitrin effectively trapped MGO with the formation of isomeric mono-MGO adducts. The effect of dry peppermint leaf extract and polyphenols in inhibiting MGO-induced glycation in vitro was comparable to that of metformin used as a positive control.

Since eriocitrin is the predominant component of *M. piperita* with anti-AGE and MGO-trapping potential, and available clinical studies support its efficacy in reversing prediabetes, the use of peppermint leaf in this regard may be warranted. However, these experiments were conducted in vitro, so the results presented here require corroboration in further in vivo studies. Nevertheless, at the current research stage, we can unequivocally confirm that the antiglycation and MGO scavenging activity of peppermint leaf and its polyphenols deserve notice.

## Figures and Tables

**Table 1:** Chemical profile of peppermint leaf dry extract confirmed by UHPLC-ESI-MS.

No.	t_R_ (min)	[M-H]^−^ (*m/z*)	MS/MS (*m/z*)	Compound	Reference
*Phenolic acids—caffetannins*					
1.	9.23	179.0355	135	caffeic acid	Stan.
2.	11.30	537.1044	493, 295, 185	lithospermic acid (lithospermic acid a)	Stan.
3.	12.85	359.0778	197, 179, 161, 135	rosmarinic acid	Stan.
4.	13.32	717.1457	519, 339, 321, 295	lithospermic acid b (salvianolic acid b)	[15]
*Flavonoids*					
5.	11.12	595.1673	287	eriodictyol-7-*O*-rutinoside (eriocitrin)	Stan.
6.	11.44	593.1508	285	luteolin-7-*O*-rutinoside (scolymoside)	Stan.
7.	11.45	449.1089	287	eriodictyol-7-*O*-β-glucoside	[15]
8.	11.73	447.0943	285	luteolin-7-*O*-β-glucoside	Stan.
9.	11.76	463.0863	287	eriodictyol-7-*O*-β-glucuronoside	[15]
10.	11.79	461.0734	285	luteolin-7-*O*-β-glucuronoside	Stan.
11.	11.98	579.1710	271	naringenin-7-*O*-rutinoside (narirutin)	Stan.
12.	12.16	577.1556	269	apigenin-7-*O*-rutinoside (isorhoifolin)	Stan.
13.	12.45	609.1816	301	hesperetin-7-*O*-rutinoside (hesperidin)	Stan.
14.	12.48	607.1667	299	diosmetin-7-*O*-rutinoside (diosmin)	Stan.
15.	12.67	445.0775	269	apigenin-7-*O*-β-glucuronoside	[15]
16.	14.43	287.0565	-	eriodictyol	Stan.
17.	14.82	285.0406	-	luteolin	Stan.
18.	16.13	269.0449	-	apigenin	Stan.
19.	16.37	301.0723	-	hesperetin	Stan.

Stan., authentic standard; tR, retention time; [M-H]^−^, pseudomolecular ion.

**Table 2:** Content of key polyphenols in peppermint leaf dry extract determined by HPLC-DAD (as heat-map).

Compound	M.W.	Content		SD	Percentage of Compound	
	g/mol	mg/g	μM/g	%	% (for mg/g)	% (for μM/g)
Caffeic acid	180.16	0.52	2.89	1.9	0.1	0.3
Rosmarinic acid	360.31	57.81	160.45	3.3	11.8	17.7
Lithospermic acid	538.46	8.29	15.40	2.4	1.7	1.7
Eriodictyol-7-*O*-rutinoside	596.54	285.37	478.38	3.4	58.1	52.9
Luteolin-7-*O*-rutinoside	594.52	78.52	132.07	3.5	16.0	14.6
Luteolin-7-*O*-β-glucoside	448.38	0.03	0.00	0.0	0.0	0.0
Luteolin-7-*O*-β-glucuronoside	462.36	27.56	59.61	3.7	5.6	6.6
Naringenin-7-*O*-rutinoside	580.54	1.24	2.14	1.1	0.3	0.2
Apigenin-7-*O*-rutinoside	578.52	3.38	5.84	2.1	0.7	0.6
Diosmin	608.55	4.74	7.79	2.3	1.0	0.9
Hesperidin	610.56	22.86	37.44	1.2	4.7	4.1
Eriodictyol	288.25	0.61	2.12	2.5	0.1	0.2
Luteolin	286.24	0.11	0.38	3.7	0.0	0.0
Apigenin	270.05	<LOQ	<LOQ	-	0.0	0.0
Hesperetin	302.28	<LOQ	<LOQ	-	0.0	0.0
Sum of phenolic acids		66.62	178.73	2.4	13.6	19.8
Sum of flavonoids		424.42	725.76	4.3	86.4	80.2
Sum of polyphenols		491.04	904.49	4.2	100.0	100.0

*n* = 6, for higher values the color is more intense; <LOQ, below the limit of quantification.

**Table 3:** Adducts of methylglyoxal with selected standards and polyphenols identified in peppermint leaf dry extract.

Compound	Pseudomolecular Ions of Adducts, *m*/*z* [Da]			
	Mono-MGO	tR [min]	Di-MGO	tR [min]
	[M+72-H]^−^		[M+144-H]^−^	
Apigenin	341.0671	28.57	413.0877	25.98
	341.0668	28.03		
Luteolin	357.0615	26.23	429.0826	24.22
	357.0615	25.97		
Luteolin-7-*O*-rutinoside	-	-	-	-
Luteolin-7-*O*-β-glucuronoside	-	-	-	-
Luteolin-7-*O*-β-glucoside	-	-	-	-
Hesperetin	373.0942	28.70	445.1126	25.33
	373.0941	28.76		
Eriodictyol	359.0772	21.48	-	-
	359.0771	21.57		
	359.0777	22.46		
	359.0775	24.67		
Eriocitrin	667.1876	19.21	-	-
	667.1874	20.59		
	667.1873	21.40		
	667.1877	24.23		
Rosmarinic acid	-	-	-	-
Peppermint leaf dry extract—Eriocitrin	667.1876	19.21	-	-
	667.1874	20.59		
	667.1873	21.40		
	667.1877	24.23		

- no adducts.

## Data Availability

Data supporting reported results are available from the corresponding author.

## Supplementary Materials

The following supporting information can be downloaded at: https://www.mdpi.com/article/10.3390/molecules28062865/s1. Figures: Figure S1. MS spectra of luteolin authentic standard; Figure S2. MS spectra of luteolin-MGO mono-adduct 1; Figure S3. MS spectra of luteolin-MGO mono-adduct 2; Figure S4. MS spectra of luteolin-MGO di-adduct; Figure S5. MS spectra of apigenin authentic standard; Figure S6. MS spectra of apigenin-MGO mono-adduct 1; Figure S7. MS spectra of apigenin-MGO mono-adduct 2; Figure S8. MS spectra of apigenin-MGO di-adduct; Figure S9. MS spectra of eriocitrin authentic standard; Figure S10. MS spectra of eriocitrin and eriocitrin-MGO mono-adduct 1; Figure S11. MS spectra of eriocitrin and eriocitrin-MGO mono-adduct 2; Figure S12. MS spectra of eriocitrin and eriocitrin-MGO mono-adduct 3; Figure S13. MS spectra of eriocitrin and eriocitrin-MGO mono-adduct 4.

## Sample Availability

Samples of the compounds will be available by arrangement with the I.F.
