# Supplementary material for: In Vitro Antiglycation and Methylglyoxal Trapping Effect of Peppermint Leaf (Mentha × piperita L.) and Its Polyphenols
Source: Molecules. 2023 Mar 22;28(6):2865. doi: 10.3390/molecules28062865 (PMC10056224; doi:10.3390/molecules28062865)
Supplement: Supplementary file 1 [file molecules-28-02865-s001.zip › molecules-2262178-supplementary.pdf]

Supplementary data to:

"In vitro antiglycation and methylglyoxal trapping effect of peppermint leaf (Mentha × piperita L.) and its polyphenols"

Izabela Fecka, Katarzyna Bednarska, Adam Kowalczyk

Department of Pharmacognosy and Herbal Medicines, Faculty of Pharmacy, Wroclaw Medical University, ul. Borowska 211, 50-556 Wroclaw, Poland

List of Figures:

Figure S1. MS spectra of luteolin authentic standard.

Figure S2. MS spectra of luteolin-MGO mono-adduct 1.

Figure S3. MS spectra of luteolin-MGO mono-adduct 2.

Figure S4. MS spectra of luteolin-MGO di-adduct.

Figure S5. MS spectra of apigenin authentic standard.

Figure S6. MS spectra of apigenin-MGO mono-adduct 1.

Figure S7. MS spectra of apigenin-MGO mono-adduct 2.

Figure S8. MS spectra of apigenin-MGO di-adduct.

Figure S9. MS spectra of eriocitrin authentic standard.

Figure S10. MS spectra of eriocitrin and eriocitrin-MGO mono-adduct 1.

Figure S11. MS spectra of eriocitrin and eriocitrin-MGO mono-adduct 2.

Figure S12. MS spectra of eriocitrin and eriocitrin-MGO mono-adduct 3.

Figure S13. MS spectra of eriocitrin and eriocitrin-MGO mono-adduct 4.

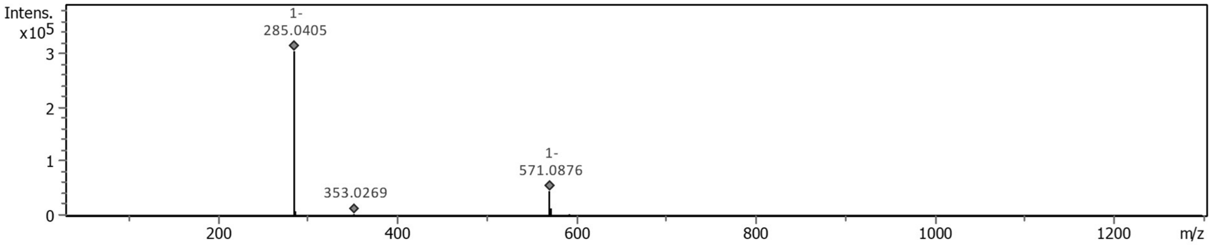

Figure S1. MS spectra of luteolin authentic standard.

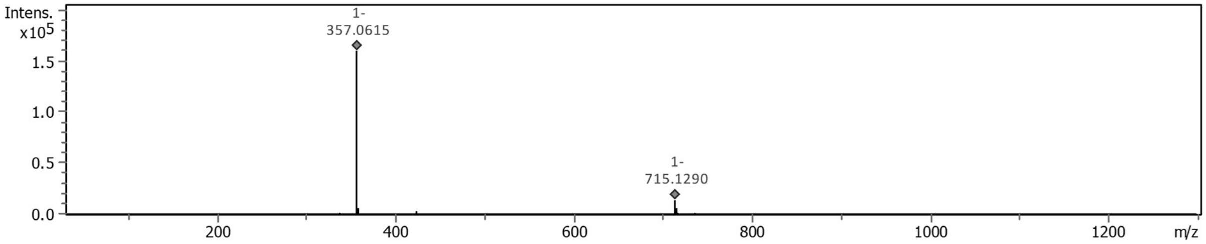

Figure S2. MS spectra of luteolin-MGO mono-adduct 1.

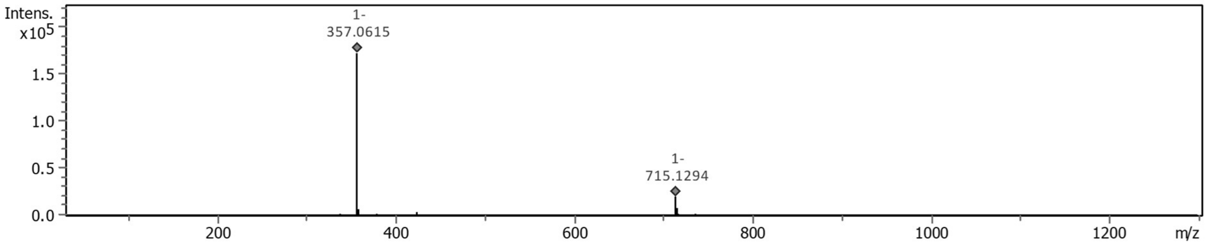

Figure S3. MS spectra of luteolin-MGO mono-adduct 2.

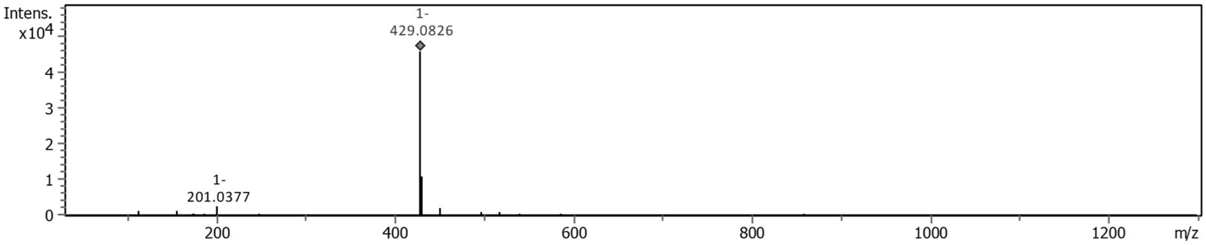

Figure S4. MS spectra of luteolin-MGO di-adduct.

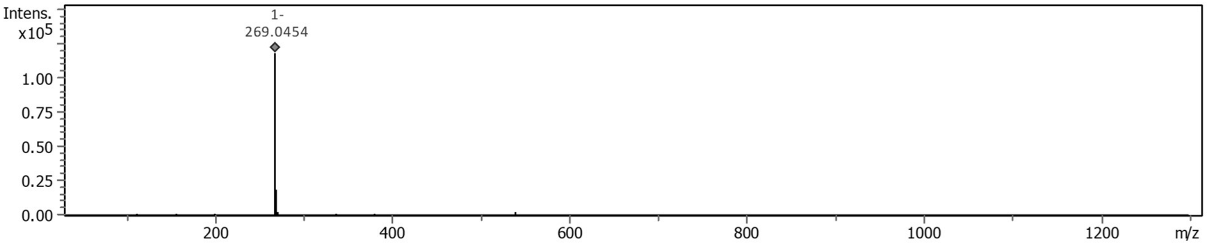

Figure S5. MS spectra of apigenin authentic standard.

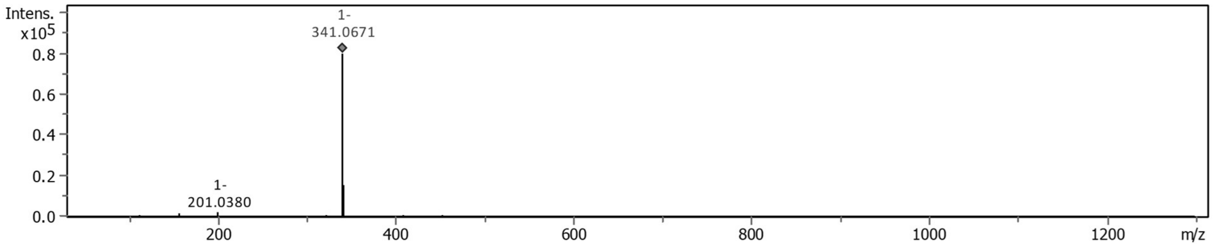

Figure S6. MS spectra of apigenin-MGO mono-adduct 1.

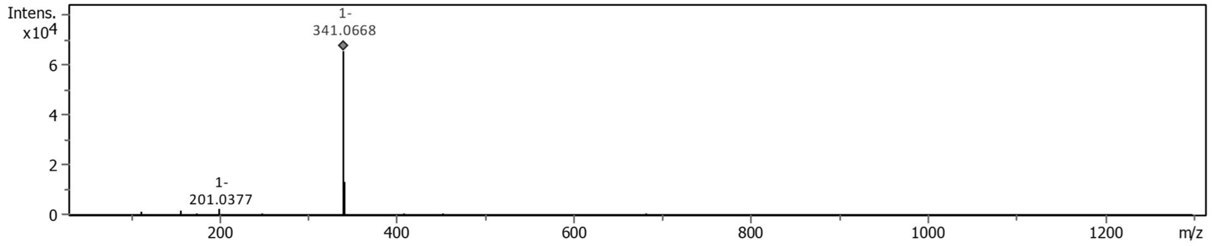

Figure S7. MS spectra of apigenin-MGO mono-adduct 2.

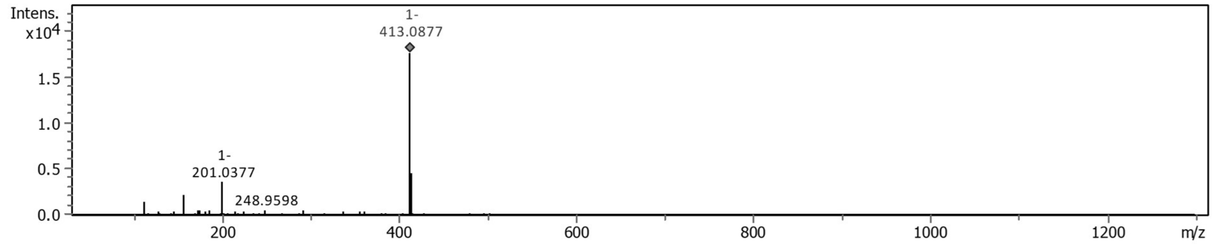

Figure S8. MS spectra of apigenin-MGO di-adduct.

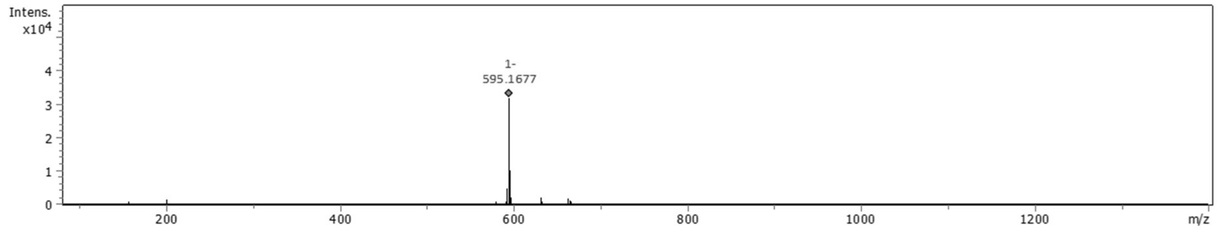

Figure S9. MS spectra of eriocitrin authentic standard.

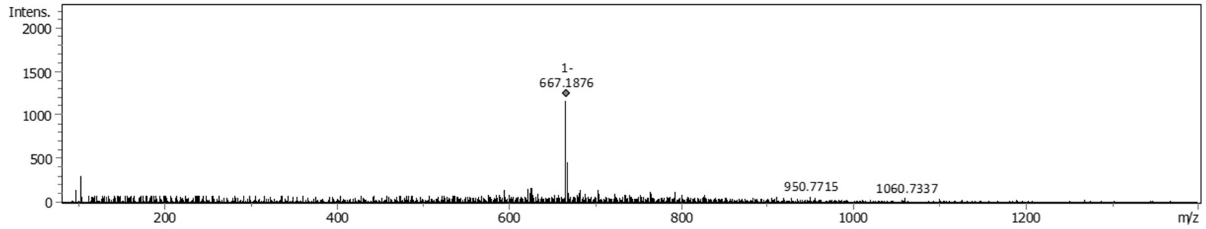

Figure S10. MS spectra of eriocitrin and eriocitrin-MGO mono-adduct 1.

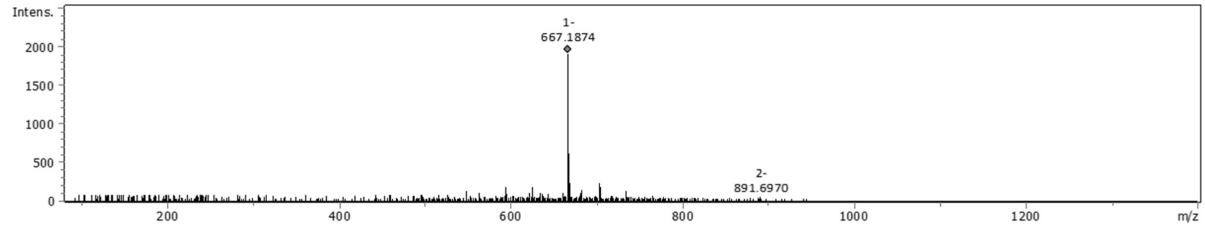

Figure S11. MS spectra of eriocitrin and eriocitrin-MGO mono-adduct 2.

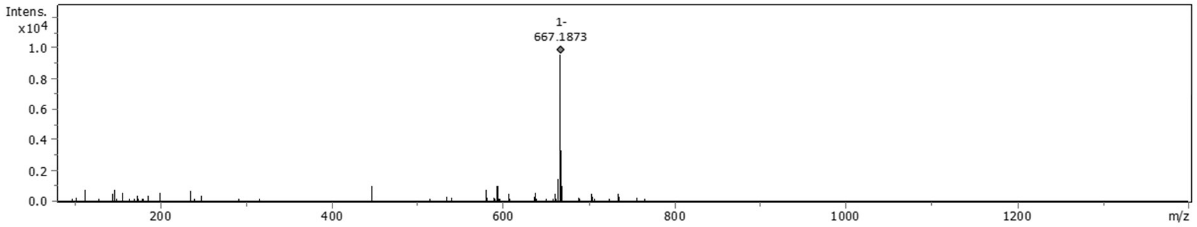

Figure S12. MS spectra of eriocitrin and eriocitrin-MGO mono-adduct 3.

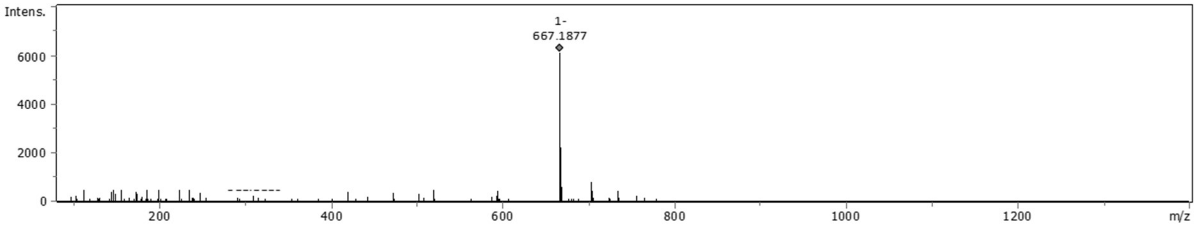

Figure S13. MS spectra of eriocitrin and eriocitrin-MGO mono-adduct 4.
